# Engineering of the glucose uptake system to increase 2,4-Dihydroxybutyric acid production in *Escherichia coli*

**DOI:** 10.1016/j.mec.2026.e00276

**Published:** 2026-04-15

**Authors:** T.A. Stefanie Nguyen, Ceren Alkim, Nadine Ihle, Thomas Walther, Cláudio J.R. Frazão

**Affiliations:** a Chair of Bioprocess Engineering, Institute of Natural Materials Technology, TU Dresden, Bergstraße 120, 01062, Dresden, Germany; b Toulouse Biotechnology Institute, UMR INSA-CNRS5504 and UMR INSA-INRAE 792, 135 avenue de Rangueil, 31077, Toulouse, France

**Keywords:** 2,4-Dihydroxybutyric acid, *Escherichia coli*, Metabolic engineering, Phosphotransferase system (PTS), Galactose permease (GalP), ^13^C metabolic flux analysis

## Abstract

2,4-Dihydroxybutyric acid (DHB) is a promising C_4_ platform compound for the synthesis of methionine analogues and biodegradable polymers. However, aerobic DHB production from glucose in *Escherichia coli* involves transient acetate overflow prior to product synthesis, which could be challenging for process scalability.

Therefore, we engineered *Escherichia coli* K-12 MG1655 for optimized DHB production by replacing the phosphotransferase system mediated glucose uptake with the galactose permease GalP, coupled to ATP-dependent phosphorylation via endogenous glucokinase. In combination with targeted deletions of malate- and fumarate-consuming reactions, we obtained a strain with enhanced flux through the tricarboxylic acid (TCA) cycle and pentose phosphate pathway leading to improved NADPH availability and increased anaplerotic activity, as revealed by ^13^C metabolic flux analyses. Deletion of the *mdh* gene encoding for the cytosolic malate dehydrogenase further promoted DHB formation. The resulting strain achieved DHB yields up to 0.20 mol mol^−1^ (2.43 g L^−1^), a 4-fold increase compared to the wildtype background (0.05 mol mol^−1^, 0.60 g L^−1^), under aerobic conditions while suppressing acetate formation.

Together, these results demonstrate that GalP-mediated glucose uptake and engineering of the TCA cycle provide a robust metabolic framework for efficient DHB biosynthesis and establish a foundation for further process and pathway development.

## Highlights

• Replacement of PTS by GalP improves DHB production.
• TCA cycle engineering increases DHB yield.
• ^13^C-MFA reveals altered central carbon flux distribution.
• Malate node controls flux toward DHB synthesis.
• Maximum DHB yield of 0.20 mol mol^−1^ achieved.

## 1 Introduction

2,4-Dihydroxybutyric acid (DHB) is an emerging platform chemical that can serve as a precursor for the methionine analogue 2-hydroxy-4-(methylthio)butyrate, for plastic intermediates such as 1,3-propanediol (Frazão et al., 2019) or 1,2,4-butanetriol (Li et al., 2014), or for the synthesis of novel biodegradable polymers (François, 2023). Moreover, DHB can be biologically produced not only from glucose (Walther et al., 2017, 2018) but also from C_2_ substrates such as ethylene glycol or glycolaldehyde (Frazão et al., 2023), all of which can be derived from waste carbon streams. These alternative feedstocks highlight the broader relevance of DHB as a value-added intermediate in the context of a circular bioeconomy. Understanding and optimizing DHB production from glucose therefore provides a foundation for the development of efficient production processes from alternative carbon sources.

**Fig. 1:**
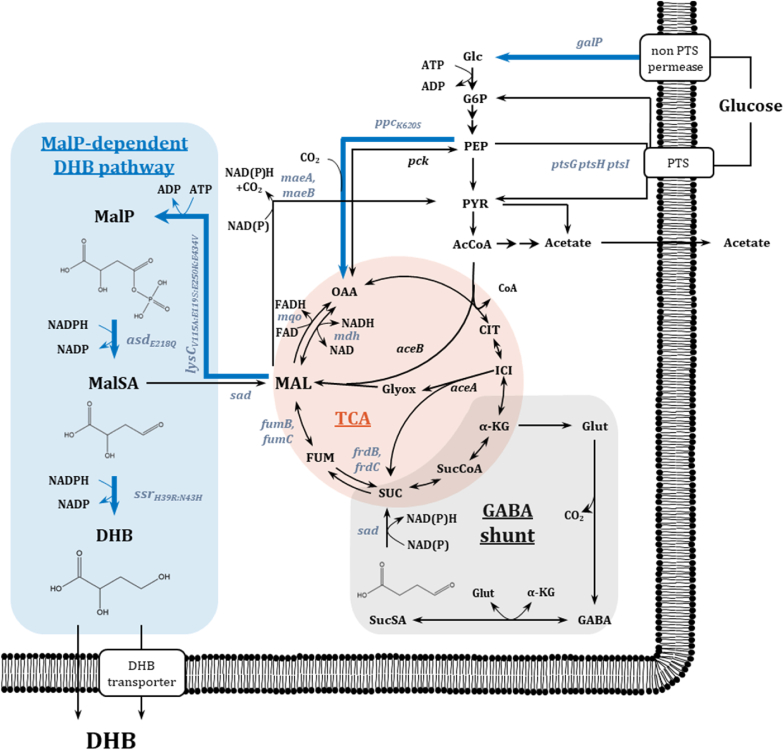
Aerobic synthesis of 2,4-Dihydroxybutyric acid (DHB) from glucose via the malyl-phosphate (MalP) and relevant metabolic pathways. The synthetic DHB pathway links the natural metabolite malate to DHB via expression of the genes *lysC*_*V115A:E119S:E250K:E434V*_ from *Escherichia coli*, *asd*_*E218Q*_ from *Bacillus subtilis* and *ssr*_*H39R:N43H*_ from *Metallosphaera sedula*. Targeted genes for strain optimization are depicted in grey-blue. Blue bold arrows show overexpressed genes. Glucose uptake via non-PTS permease is followed by phosphorylation reaction through endogenous ATP-dependent glucokinase enzyme. Abbreviations: AcCoA – Acetyl-CoA; AcP – Acetyl phosphate; *α*-KG – *α*-Ketoglutarate; ASP – Aspartate; CIT – Citrate; FUM – Fumarate; GABA – *γ*-aminobutyric acid; Glc – Glucose; G6P – Glucose-6-phosphate; Glut – Glutamate; Glyox – Glyoxylate; ICI – Isocitrate; DHB – 2,4-Dihydroxybutyric acid; MAL – Malate; MalP – Malyl phosphate; MalSA – Malate semialdehyde; OAA – Oxaloacetate; PEP – Phosphoenolpyruvate; PTS – Phosphotransferase system; PYR – Pyruvate; SUC – Succinate; SucCoA – Succinyl-CoA; SucSA – Succinate semialdehyde; TCA – Tricarboxylic acid cycle.

The most studied synthetic routes toward DHB production in *Escherichia coli* originate either from the native intermediate (L)-homoserine (Walther et al., 2018; Frazão et al., 2018; Liu et al., 2022; Zhang et al., 2025; Ihle et al., 2025) or from (L)-malate (Walther et al., 2017; Nguyen et al., 2025). In our previous work (Nguyen et al., 2025), we optimized the productivity of strains operating the malyl phosphate (MalP) pathway by introducing the malate-insensitive phosphoenolpyruvate carboxylase variant Ppc_K620S_ and deleting the NAD-dependent succinic semialdehyde dehydrogenase activity (encoded by *sad*) (Fig. 1). Despite this improvement, DHB synthesis was usually preceded by a distinct phase of acetate secretion and subsequent reassimilation, suggesting a complex metabolic coupling between acetate metabolism and DHB formation. Such dynamic flux changes can make process control and scale-up challenging, since the onset of DHB synthesis appears to depend on the transition from overflow metabolism to acetate reutilization. Therefore, we aimed to develop suitable chassis strains capable of efficient DHB production without acetate accumulation, forming the basis for further pathway and process development.

Phosphotransferase system (PTS)-mediated glucose uptake often causes acetate overflow through high glycolytic flux (Millard et al., 2021; Chubukov et al., 2014; Vemuri et al., 2006), while simultaneously forcing the conversion of phosphoenolpyruvate (PEP) to pyruvate, as PEP serves as the phosphate donor during glucose transport. But PEP also represents a key entry point for anaplerotic reactions that replenish oxaloacetate and thereby sustain TCA cycle activity. Its consumption by the PTS can therefore restrict anaplerotic flux into the TCA cycle. Under conditions of glucose excess, this imbalance becomes more pronounced, as high glycolytic flux exceeds the reduced capacity of the TCA cycle, promoting carbon loss toward pyruvate and downstream overflow pathways. To circumvent these limitations, the native PTS can be replaced with the ATP-dependent glucose uptake via GalP, which actively imports the sugar through a proton gradient, and glucokinase (Henderson et al., 1977). This system has previously been applied for the anaerobic overproduction of (L)-malate (Zhang et al., 2011), where additional deletions of malic enzymes, fumarate reductase, and fumarases were required to channel carbon flux toward the target product. However, DHB biosynthesis depends on both ATP and NADPH, and it is therefore more compatible with aerobic metabolism, where cofactor regeneration and energy supply are improved.

Guided by these insights, we engineered *E. coli* strains by enabling GalP-mediated glucose uptake, and by inactivating malate- and fumarate-consuming reactions. The resulting chassis strains were evaluated based on their aerobic DHB production via the MalP-dependent pathway (Fig. 1), which involves phosphorylation of malate to MalP followed by its reduction to malate semialdehyde and ultimately DHB. Additionally, we applied ^13^C-metabolic flux analysis (MFA) to quantify intracellular carbon redistribution among glycolytic, anaplerotic, and TCA cycle routes. Building on engineering concepts from our previous studies (Ihle et al., 2025; Nguyen et al., 2025), including modification of *sad*, *ppc*, *pntAB*, *mqo*, and *mdh*, we explored further metabolic requirements for optimizing DHB production in the newly constructed chassis strains.

## 2 Materials and methods

### 2.1 Chemicals and reagents

All chemicals were purchased from Sigma-Aldrich (Darmstadt, Germany) unless stated otherwise. The kits for plasmid DNA isolation and PCR clean-up were purchased from New England Biolabs (Frankfurt am Main, Germany) and used according to the instructions of the manufacturer. PCR reactions were performed with Q5 DNA polymerase (New England Biolabs) for all subcloning experiments. Dream Taq PCR Master Mix (ThermoFisher Scientific, Waltham, MA, USA) was used for colony PCR. All primers were synthesized by Sigma-Aldrich.

### 2.2 Strain construction

All strains used in this study (Table 1) derive from the parental strain *E. coli* K-12 substr. MG1655 (ATCC 47076). The chromosomal deletion of the genes *ptsI, maeA, maeB, fumC, sad, mdh* and *mqo* was achieved by P1vir phage transduction (Lennox, 1955). Phage lysates were created from single knock-out strains of the Keio collection (Baba et al., 2006). For the deletion of *frdBC*, replacement of *ppc* gene Δ*ppc::ppc*_*K620S*_ and exchange of endogenous promoter controlling expression of *ppc* gene ΔP_ppc_::P_proA_ (Davis et al., 2011), phage lysates were created from MG1655-derived donor strains kindly provided by Prof. Jean Marie François, INSA Toulouse. Phage lysates for exchange of endogenous promoter controlling expression of *galP* gene ΔP_pntAB_::P_proD_ were used from Ihle et al. (2025) (Table 1).

**Table 1:** Host strains, all derived from *E. coli* K-12 MG1655, and plasmids used in this study.

Host strain	Genotype	Reference
WT	*E. coli* K-12 MG1655 (*F*^*−*^*λ*^*-*^*ilvG- rfb-50 rph-1*)	ATCC
GalP0	*ΔptsI*	This study
GalP1	ΔP_galP_::P_proD_*ΔptsI*	This study
GalP2	ΔP_galP_::P_proD_	This study
GalP3	ΔP_galP_::P_proD_*ΔptsI ΔmaeA*	This study
GalP4	ΔP_galP_::P_proD_*ΔptsI ΔmaeA ΔmaeB*	This study
GalP5	ΔP_galP_::P_proD_*ΔptsI ΔmaeA ΔmaeB ΔfrdBC*	This study
GalP6	ΔP_galP_::P_proD_*ΔptsI ΔmaeA ΔmaeB ΔfrdBC ΔfumB*	This study
GalP7	ΔP_galP_::P_proD_*ΔptsI ΔmaeA ΔmaeB ΔfrdBC ΔfumB ΔfumC*	This study
Frd1	*ΔfrdBC*	This study
GalP8	ΔP_galP_::P_proD_*ΔfrdBC*	This study
GalP9	ΔP_galP_::P_proD_*ΔfrdBC ΔmaeA ΔmaeB*	This study
GalP10	ΔP_galP_::P_proD_*ΔfrdBC ΔmaeA ΔmaeB ΔfumB*	This study
GalP11	ΔP_galP_::P_proD_*ΔfrdBC ΔmaeA ΔmaeB ΔfumB ΔfumC*	This study
Sad1	*Δsad*	Nguyen et al. (2025)
GalP12	ΔP_galP_::P_proD_*ΔptsI ΔmaeA ΔmaeB ΔfrdBC ΔfumB ΔfumC Δsad::kan*	This study
GalP13	ΔP_galP_::P_proD_*ΔptsI ΔmaeA ΔmaeB ΔfrdBC ΔfumB ΔfumC Δppc::ppc*_*K620S*_ ΔP_ppc_::P_proA-kan_	This study
GalP14	ΔP_galP_::P_proD_*ΔptsI ΔmaeA ΔmaeB ΔfrdBC ΔfumB ΔfumC* ΔP_pntAB_::P_proD-kan_	This study
GalP15	ΔP_galP_::P_proD_*ΔptsI ΔmaeA ΔmaeB ΔfrdBC ΔfumB ΔfumC Δmqo::kan*	This study
GalP16	ΔP_galP_::P_proD_*ΔptsI ΔmaeA ΔmaeB ΔfrdBC ΔfumB ΔfumC Δmdh::kan*	This study

**Donor strain**	**Genotype**	**Reference**
bWL1043	*Δppc::ppc*_*K620S*_ ΔP_ppc_::P_proA-kan_	INSA Toulouse
EcHS7	*ΔthrB ΔmetA ΔldhA* ΔP_pntAB_::P_proD-kan_	Ihle et al. (2025)

**Plasmid**	**Relevant characteristics**	**Reference**
pKD4	*R6K γ* ori, FRT-flanked Kan^R^, and Amp^R^	Datsenko and Wanner (2000)
pKD46	*pSC101* ori, *araC-ParaB*, λ-Red recombinase system (*γ, β, exo, λ tL3* terminator), and Amp^R^	Datsenko and Wanner (2000)
pCP20	*pSC101* ori (temperature sensitive), Amp^R^, Cm^R^, Flp	Cherepanov and Wackernagel (1995)
pZA33	*p15A* ori, Cm^R^, P_A1lacO-1_ promoter	Expressys
pZA33-DHBop-ppc_K620S_	pZA33 derivative harboring *Ec.ppc*_*K620S*_ and the DHB operon: *Ec.lysC*_*V115A:E119S:E250K:E434V*_*; Bs.asd*_*E218Q*_*; Ms.ssr*_*H39R:N43H*_	Nguyen et al. (2025)
pZA33-DHBop	pZA33 derivative harboring the DHB operon	Nguyen et al. (2025)

The gene *fumB* was deleted by the method of Datsenko and Wanner (2000). Briefly, the kanamycin resistance cassette (kan) flanked with the Flp recombinase recognition target (FRT) sites (FRT-kan-FRT) was amplified by PCR from the pKD4 plasmid (Table 1). The primers used additionally introduced 50 bp sequence homologous to the regions flanking the gene *fumB*. The resulting PCR product was subsequently treated with DpnI, purified and used to transform into the recipient strain harboring the pKD46 plasmid (Table 1), which expresses the λ-Red recombinase genes.

For both methods, the positive clones were selected on LB (5 g L^−1^ yeast extract, 10 g L^−1^ tryptone and 10 g L^−1^ NaCl) agar plates (15 g L^−1^ agar-agar, Carl Roth) supplemented with 50 mg L^−1^ kanamycin sulfate and verified by colony PCR with locus-specific primers (Supplementary File 1 Table S6). Before each additional round of gene deletion, the kanamycin resistance cassette was excised by the FLP recombinase encoded from the pCP20 plasmid (Cherepanov and Wackernagel, 1995), yielding antibiotic-sensitive strains. For DHB production, all strains were chemically transformed (Green and Sambrook, 2012) with either one of our previously reported production plasmids pZA33-DHBop-ppc_K620S_ or pZA33-DHBop (Table 1) as described in relevant sections (Nguyen et al., 2025).

### 2.3 Cell cultivation conditions

All cultivations were performed in an orbital shaker set at 37 °C and at a shaking frequency of 220 rpm (Ecotron, Infors, Switzerland). M9 medium was used as the minimal medium. One liter of M9 medium contains: 18 g Na_2_HPO_4_·12H_2_O, 3 g KH_2_PO_4_, 0.5 g NaCl, 2 g NH_4_Cl, 0.5 g MgSO_4_·7H_2_O, 0.015 g CaCl_2_·2H_2_O, 0.010 g FeCl_3_, 0.012 g thiamine HCl and 1 ml of trace element solution (containing per liter: 0.04 g Na_2_EDTA·2H2O, 0.18 g CoCl_2_·6H2O, ZnSO_4_·7H2O, 0.04 g Na_2_MoO_4_·2H_2_O, 0.01 g H_3_BO_3_, 0.12 g MnSO_4_·H2O, 0.12 g CuCl_2_·H_2_O), 35 mg L^−1^ chloramphenicol and was buffered with 100 mM 3-(N-morpholino)-propanesulfonic acid (MOPS) that was adjusted to pH 7 with KOH.

The first pre-culture (PC1) was inoculated with a colony from a freshly-streaked LB plate supplemented with 35 mg L^−1^ chloramphenicol in a 15 mL screw cap tube containing 3 mL LB. After 8 h of cell cultivation, 0.5 mL of PC1 was used to inoculate a second pre-culture (PC2) which was performed in a 50 mL screw cap tube containing 10 mL M9 medium supplemented with 20 g L^−1^ D-glucose. After 16 h of incubation, cells were harvested by centrifugation (5 min at 6000×*g*, room temperature) and used to inoculate the main culture (25 mL M9 medium in 250 mL baffled shake flask) to an optical density at 600 nm (OD_600_) of 0.2. At early log-phase (OD_600_ around 0.6), the expression of genes of the DHB pathway was induced by adding isopropyl-β-D-thiogalactopyranoside (IPTG) at a final concentration of 1 mM. Cell growth was monitored by measuring optical densities in a GENESYS 150 UV-Vis spectrophotometer (ThermoFisher Scientific, Waltham, MA, USA). Samples for HPLC analysis were taken by centrifugation (2 min, 16,000×*g*, room temperature) and the supernatant was stored at −20 °C until further processing.

### 2.4 HPLC analyses

For assessing DHB production, the liquid samples were analyzed by high-performance liquid chromatography (HPLC, UltiMate 3000 system, Dionex, USA) using the cation-exchange column Rezex™ RoA-organic acid H^+^ 8% (300 × 7.8 mm, Phenomenex, USA) preceded by a SecurityGuard™ guard cartridge (Carbo H^+^, 4 × 3 mm, Phenomenex, USA) heated at 80 °C. The samples were syringe-filtered (PTFE, pore size, 0.2 μm) and 20 μL were injected using 0.5 mM H_2_SO_4_ as the mobile phase and a constant flow rate of 0.5 mL min^−1^. The RI detector (ERC RefractoMax 520, 45 °C, Knauer, Germany) was used for the detection of glucose, DHB, acetate and other potential side products (fumarate, succinate, lactate, ethanol). Malate, pyruvate and formate were analyzed with the UV/Vis detector set at 210 nm (Dionex, Sunnyvale, USA). The software Chromeleon (version 7.2 SR5, Dionex) was used for the control of the system and subsequent data evaluation.

### 2.5 Metabolic flux analysis

**Cell cultivation:** 3 mL of LB was inoculated with a colony from a LB plate supplemented with 35 mg L^−1^ chloramphenicol and incubated (37 °C, 220 rpm) in a 15 mL screw cap tube (PC1). After 8 h of cultivation, 10 mL of M9 (20 g L^−1^ glucose) supplemented with 1 mM IPTG (PC2) was inoculated with 0.01 mL of PC1. After overnight incubation (16 h), the cells were harvested (5 min at 6000×*g*, room temperature). For the labeling experiment, 100 mL baffled shake flasks containing 10 mL of M9 medium with 2.5 g L^−1^ glucose (1:4 ratio of U-^13^C to 1-^13^C) and 1 mM IPTG were used. The main culture was inoculated at an initial OD_600_ of 0.02. When an OD_600_ of 1 was reached, cells were collected by centrifugation (6000×*g*, 5 min, 4 °C) and the supernatant was retained for analysis of extracellular metabolites. The cell pellet was washed twice with a solution of 0.9 % (w/v) NaCl. All supernatants and cell pellets were stored at −20 °C until further analysis.

**Analysis of extracellular metabolites:** Acetate was analyzed by HPLC as previously described. The extracellular metabolites glucose and DHB were analyzed by liquid chromatography coupled with a mass spectrometer (LC/MS) using the Thermo Scientific™ Q Exactive™ Focus device controlled by Xcalibur software (version 2.1, ThermoFisher Scientific). The column Rezex™ RoA-organic acid (same as employed for HPLC analyses) was used for metabolite separation with a solution of 0.1 % (w/v) formic acid as mobile phase and at an isocratic flow rate of 0.4 mL min^−1^. The optimal electron spray was achieved with a sheath gas flow rate of 32 arbitrary (arb.) units, auxiliary gas flow rate of 8 arb. units, sweep gas flow rate of 0 arb. units, spray voltage of −3.5 kV, capillary temperature of 250 °C and auxiliary gas temperature of 200 °C (Frazão et al., 2023; Wagner et al., 2023a).

**Analysis of proteinogenic amino acids**: Thawed cell pellets were resuspended in 250 μL of a 6 M HCl solution and incubated in an airtight glass vial for 4 h at 103 °C (UE400 Universal Oven, Memmert, Germany). Subsequently, the samples were centrifuged (16,000×*g*, 5 min) in a 2 mL reaction tube to remove cell debris. The supernatant was then transferred to a fresh reaction tube and evaporated for 4 h at 45 °C in a benchtop vacuum concentrator (CentriVap Concentrator System, Labconco, USA). Afterwards, the samples were resuspended in 250 μL deionized water.

For LC/MS analysis of the amino acid fractions, 10 μL of the resuspended sample was diluted in a matrix composed of 60 % (v/v) acetonitrile (AcN), 40 % (v/v) deionized water and 10 mM ammonium acetate (AA, pH 9.2) to a final volume of 1 mL. The liquid chromatography was performed with the Vanquish system from ThermoFisher Scientific using a SeQuant® ZIC®-pHILIC column (5 μm polymer 150 × 2.1 mm) set at a temperature of 25 °C and at a flow rate of 0.15 mL min^−1^. For optimal separation a gradient of mobile phase A (5 % AcN, 10 mM AA, pH 9.2) and B (90 % AcN, 10 mM AA, pH 9.2) was set as follows: 0 min, 95% B; 2 min, 95% B; 3 min, 89.4% B; 5 min, 89.4% B; 6 min, 83.8% B; 7 min, 83.8% B; 8 min, 78.2% B; 9 min, 78.2% B; 10 min, 55.9% B; 12 min, 55.9% B; 13 min, 27.9% B; 16 min, 27.9% B; 18 min, 0% B; 23 min, 0% B; 24 min, 95% B; 30 min, 95% B. The same electron spray settings as in the analyses of the extracellular metabolites were used (Wagner et al., 2023a).

**Calculation of fluxes:** To calculate the metabolic fluxes from the protein-bound amino acid fractions and extracellular metabolomes, the correction for natural occurring isotopes was achieved using IsoCor (Millard et al., 2012). The corrected labeling patterns of proteinogenic amino acids from two biological replicates, used for flux estimation, are provided in Supplementary File 2. The flux distribution was determined with the software *influx_s* version 5.3.0 (Sokol et al., 2012) using the provided FTBL file for *E. coli* K-12 MG1655, which defines the metabolic network and constraints for central carbon metabolism, including glycolysis, the pentose phosphate and Entner–Doudoroff pathways, the TCA cycle, anaplerotic reactions, and acetate formation. For the DHB producer strains, the model was expanded with an output reaction of DHB from malate and the glucose uptake through the GalP/Glk system without conversion of PEP to pyruvate. Furthermore, the flux from malate to pyruvate and the glucose uptake from PTS was set to zero when necessary. The flux values represent the mean of two independent biological experiments, and the reported variation reflects the deviation between these replicates and should not be interpreted as statistical confidence intervals of the model fit. The goodness-of-fit of the flux estimation was evaluated using the χ^2^ values comparing measured and simulated labeling patterns (Supplementary File 1 Table S4).

## 3 Results

### 3.1 Construction of DHB-producing strains engineered for GalP-mediated glucose uptake

**Fig. 2:**
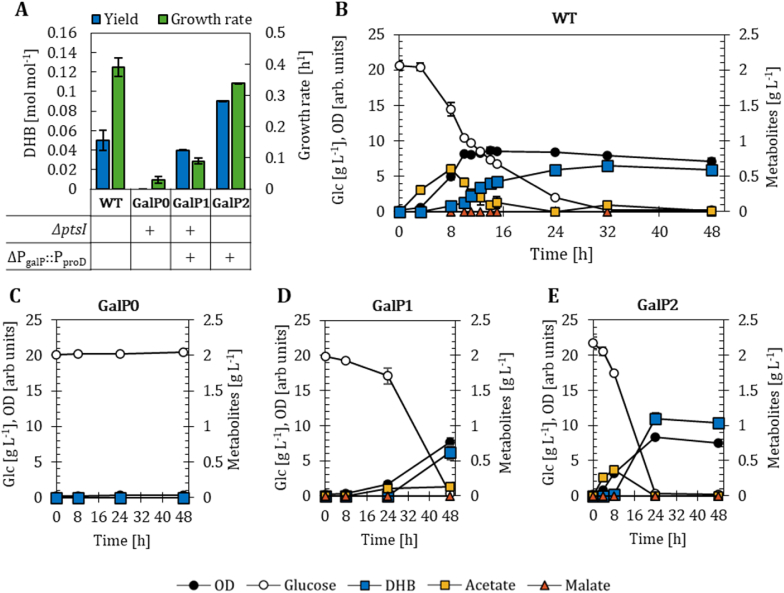
DHB yields after 48 h of cultivation and growth rates of strains engineered for GalP-mediated glucose uptake (A). Strains are harbouring the DHB production plasmid pZA33-DHBop-ppc_K620S_. Corresponding fermentation profiles in M9 minimal medium supplemented with 20 g L^−1^ of glucose are shown for: MG1655 wild type (WT, B); *ΔptsI* (GalP0, C); ΔP_galP_::P_proD_*ΔptsI* (GalP1, D); ΔP_galP_::P_proD_ (GalP2, E). In (A) deletion (Δ) of target genes is marked with a (+) sign; in (B-E) mean values are shown as colored symbols. All results correspond to the mean of at least two biological replicates with error bars representing the standard deviation of the mean (see Supplementary File 1 Table S1).

We previously showed DHB production from glucose using *E. coli* K-12 MG1655 (WT) bearing the plasmid pZA33-DHBop-ppc_K620S_ (Fig. 2A and B). The DHB operon (DHBop) encodes for the malate kinase *lysC*_*V115A:E119S:E250K:E434V*_ from *E*. *coli*, malate semialdehyde dehydrogenase *asd*_*E218Q*_ from *B*. *subtilis* and malate semialdehyde reductase *ssr*_*H39R:N43H*_ from *M*. *sedula* (Walther et al., 2017). In addition, the plasmid contains the malate-insensitive PEP carboxylase gene *ppc*_*K620S*_ from *E*. *coli* (Trichez et al., 2018). The detailed analysis of the fermentation profile of the wildtype background bearing the DHB plasmid (Fig. 2B) revealed that small amounts of acetate (∼0.5 g L^−1^) were secreted to the medium during cell growth (0-8 h). Acetate was eventually fully reconsumed in parallel with the synthesis of DHB. To prevent the futile formation of acetate, we decided to construct host strains deficient in the PTS-dependent uptake system, which has previously been shown (Fuentes et al., 2013) to support slower growth on glucose-based mineral medium while reducing or eliminating transient acetate accumulation compared to the WT host strain (Fig. 2A; Supplementary File 1 Table S1).

We started our endeavor by deleting from the DHB-producer strain the PTS enzyme I (PtsI), which is an essential part of the PTS complex and directly catalyzes the reaction from PEP to pyruvate to gain the phosphate group for the subsequent phosphorylation of glucose to glucose-6-phosphate (Chauvin et al., 1996). The new strain GalP0 grew very poorly (0.03 h^−1^, Fig. 2A) and reached a final OD_600_ of only 0.32 (Fig. 2C), which is not even one doubling of the initial OD_600_ (0.18). This confirms that the sole deletion of *ptsI* is sufficient to disrupt the PTS system. We speculate that the observed basal growth is due to residual glucose uptake through low-level expression of alternative transporters such as GalP or the mannose permease system (Steinsiek and Bettenbrock, 2012).

Next, we overexpressed the galactose permease GalP, which is a non-PTS sugar permease capable of importing glucose. The sugar is subsequently phosphorylated by the endogenously expressed ATP-dependent glucokinase Glk prior to entering the Embden-Meyerhof-Parnas pathway (Fraenkel et al., 1964). While GalP becomes the predominant glucose transporter when the PTS system is inactive (Zhang et al., 2009), its native expression level is insufficient to sustain efficient glucose uptake and growth. Therefore, we placed *galP* under control of the constitutive proD promoter (Davis et al., 2011) to ensure continuous and strong expression. The resulting strain, termed GalP1, grew at a rate of 0.09 h^−1^ (Fig. 2A–D) which is a three-fold improvement relative to strain GalP0. But despite the successful overexpression of *galP*, DHB production after 48 h of cultivation remained lower (0.04 mol mol^−1^) compared to the WT background (Fig. 2A). We further constructed the DHB-producing strain GalP2, which contains an intact PTS-uptake system and chromosomally overexpresses *galP*. While significant amounts of acetate were transiently accumulated, this strain produced DHB (0.09 mol mol^−1^; Fig. 2A and E) at the highest yield amongst the four tested strains. We therefore retained both strains – GalP1 and GalP2 (PTS-inactive and PTS-active, respectively) – as a basis to eventually improve DHB titers and yields.

### 3.2 Influence of TCA cycle deletions on DHB production in PTS-inactive strains

**Fig. 3:**
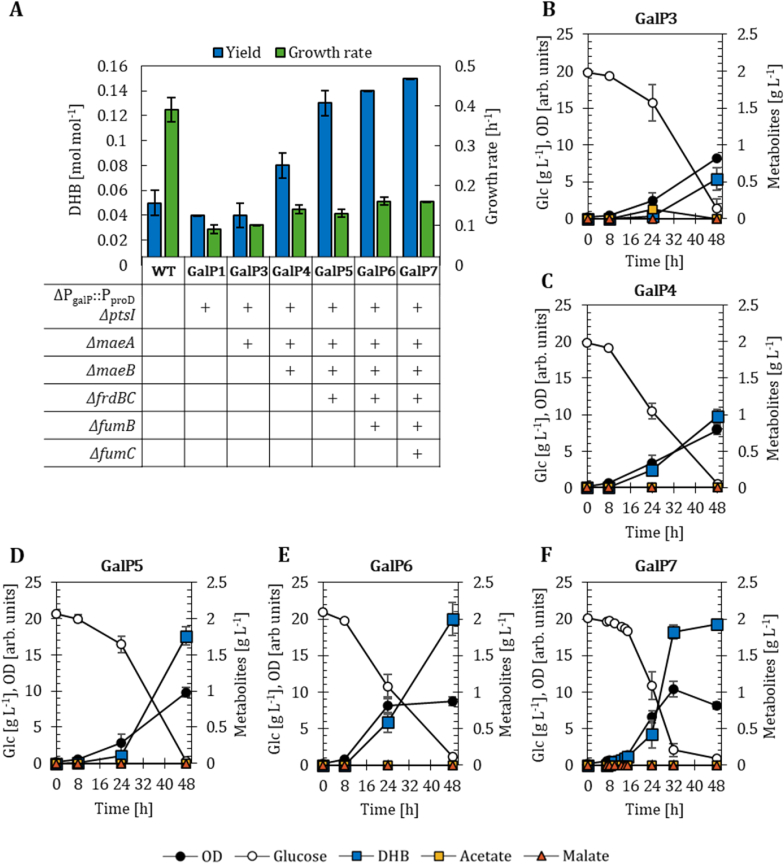
DHB yields after 48 h of cultivation and growth rates of PTS-deficient strains overexpressing the *galP* gene (A). Strains are harbouring the DHB production plasmid pZA33-DHBop-ppc_K620S_. Corresponding fermentation profiles in M9 minimal medium supplemented with 20 g L^−1^ of glucose are shown for: ΔP_galP_::P_proD_*ΔptsI ΔmaeA* (GalP3, B); ΔP_galP_::P_proD_*ΔptsI ΔmaeA ΔmaeB* (GalP4, C); ΔP_galP_::P_proD_*ΔptsI ΔmaeA ΔmaeB ΔfrdBC* (GalP5, D); ΔP_galP_::P_proD_*ΔptsI ΔmaeA ΔmaeB ΔfrdBC ΔfumB* (GalP6, E); ΔP_galP_::P_proD_*ΔptsI ΔmaeA ΔmaeB ΔfrdBC ΔfumB ΔfumC* (GalP7, F). In (A) deletion (Δ) of target genes is marked with a (+) sign; in (B-E) mean values are shown as colored symbols. All results correspond to the mean of at least two biological replicates with error bars representing the standard deviation of the mean (see Supplementary File 1 Table S1).

The PTS-inactive GalP1 strain equipped with the pZA33-DHBop-ppc_K620S_ plasmid produced only very low levels of acetate. Building on this advantageous behavior, we set out to improve DHB production by inactivating potential malate-consuming reactions (Zhang et al., 2011). We sequentially inactivated the two endogenous malic enzymes (MaeA, MaeB) in the GalP1 strain. While the deletion of *maeA* (GalP3; Fig. 3A and B) was not beneficial, the inactivation of both enzymes (GalP4; Fig. 3A and C) raised the DHB yield to 0.08 mol mol^−1^ (Supplementary File 1 Table S1). This indicates that at least one malic enzyme was active in PTS-deficient strains. The deletion of both malic enzymes also abolished acetate secretion and no other byproducts from the TCA cycle (malate, fumarate, succinate) or fermentative pathways (lactate, ethanol) were observed in the HPLC analysis.

As the inactivation of the fumarate reductase Frd has previously been shown to be essential for the anaerobic production of (L)-malate (Zhang et al., 2011), we examined whether this observation could also be reproduced under aerobic conditions. We therefore inactivated the *frdBC* genes in the GalP4 host strain obtaining strain GalP5 to further increase the malate pool and therefore DHB production. Indeed, the DHB yield improved 1.7-fold (0.13 mol mol^−1^, Fig. 3A and D) compared to strain GalP4. Even though the fumarate reductase is mainly active under anaerobic conditions (Jones and Gunsalus, 1987), our results suggest that the enzyme complex is also functional under aerobic conditions. Because the inactivation of Frd had a positive impact on aerobic DHB production, we next targeted the deletion of fumarases FumB and FumC, which catalyze the interconversion of malate and fumarate either under anaerobic or oxidative stress conditions (Tseng et al., 2001). We decided not to delete aerobic fumarase FumA to not impair the oxidative TCA cycle. The sequential deletion of *fumB* and *fumC* genes (resulting in GalP6 and GalP7, respectively) improved DHB yields up to 0.15 mol mol^−1^ (Fig. 3A and E-F). A detailed analysis of the GalP7 fermentation profile (Fig. 3F) revealed that DHB production initiates at the start of induction and occurs mainly during the growth phase, unlike the WT background where DHB synthesis is delayed until acetate is consumed. Acetate or other fermentative products, as well as secretion of TCA cycle intermediates, could not be detected with HPLC.

Overall, these results show that inactivation of malate- and fumarate-consuming reactions improves DHB production in the PTS-deficient GalP1 background.

### 3.3 Influence of deletions of TCA cycle enzymes on DHB production in PTS-active strains

Since the inactivation of competing malate-consuming reactions had a strong impact in the PTS-deficient background (GalP1-derived hosts), we next examined whether identical results could be achieved in strains that retain PTS-mediated glucose uptake while overexpressing *galP*. We therefore selected GalP2 for further strain engineering. Compared to the GalP1 background, GalP2 already exhibited higher DHB production after 48 h of cultivation when both strains express the pZA33-DHBop-ppc_K620S_ plasmid.

**Fig. 4:**
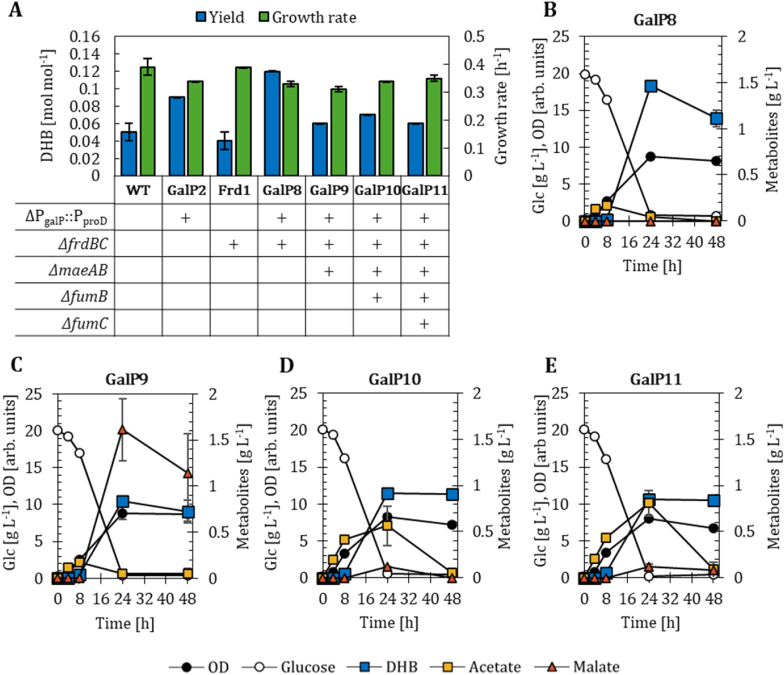
DHB yields after 24 h of cultivation and growth rates of PTS-active strains overexpressing the *galP* gene (A). Strains are harbouring the DHB production plasmid pZA33-DHBop-ppc_K620S_. Corresponding fermentation profiles in M9 minimal medium supplemented with 20 g L^−1^ of glucose are shown for_S_: ΔP_galP_::P_proD_*ΔfrdBC* (GalP8, B); ΔP_galP_::P_proD_*ΔfrdBC ΔmaeA ΔmaeB* (GalP9, C); ΔP_galP_::P_proD_*ΔfrdBC ΔmaeA ΔmaeB ΔfumB* (GalP10, D); ΔP_galP_::P_proD_*ΔfrdBC ΔmaeA ΔmaeB ΔfumB ΔfumC* (GalP11, E). In (A) deletion (Δ) of target genes is marked with a (+) sign; in (B-E) mean values are shown as colored symbols. All results correspond to the mean of at least two biological replicates with error bars representing the standard deviation of the mean (see Supplementary File 1 Table S2).

Because the inactivation of Frd was the most beneficial modification in the PTS-deficient strains, *frdBC* was first deleted in the GalP2 strain yielding the new host strain GalP8. When equipped with the pZA33-DHBop-ppc_K620S_ plasmid, GalP8 showed improved DHB production after 24 h of cultivation with a yield of 0.12 mol mol^−1^ compared to host GalP2 (Fig. 4A; Supplementary File 1 Table S2). But between 24 and 48 h part of the DHB was reconsumed from 1.46 to 1.11 g L^−1^ (Fig. 4B), which was not observed for the GalP2 background. Therefore, we continued to compare DHB yields after 24 h of cultivation. At the same time, we evaluated DHB production in a strain only devoid of the *frdBC* genes (strain Frd1, Fig. 4A). However, no improvement in DHB production compared to WT was observed, which suggests a potential link between GalP-mediated glucose uptake and Frd activity or the expression of corresponding genes.

We next inactivated both malic enzymes MaeA and MaeB from the DHB-producer strain GalP8, yielding strain GalP9. Unexpectedly, DHB production decreased to 0.07 mol mol^−1^ (Fig. 4A), while 1.61 g L^−1^ malate accumulated after 24 h (Fig. 4C). This suggests that the conversion of malate to DHB became limiting under these conditions—a phenomenon absent in the PTS-deficient background. Finally, the fumarase-encoding genes *fumB* and *fumC* were sequentially deleted in GalP9, resulting in GalP10 (*ΔfumB*) and GalP11 (*ΔfumB ΔfumC*). The inactivation of FumB did not increase DHB yield, though malate decreased to 0.12 g L^−1^. Similarly, deletion of *fumC* provided no additional benefit. In both strains, acetate accumulated transiently (0.81 g L^−1^ after 24 h) but declined to 0.1 g L^−1^ by the end of cultivation (Fig. 4D and E).

Together, these results show that deletions of malate- and fumarate-associated reactions also influence PTS-active strains, confirming that these pathways were activated when GalP-mediated glucose uptake is increased. While Frd inactivation modestly enhanced DHB production, the additional deletions of malic enzymes or fumarases (FumB, FumC) did not further improve yields. However, in contrast to the PTS-deficient background, these modifications did not result in a consistent improvement of DHB production and were associated with transient acetate accumulation, malate secretion, and partial reconsumption of DHB. In comparison, the PTS-deficient GalP7 strain showed superior DHB production performance. Therefore, GalP7 was selected for subsequent ^13^C-MFA to investigate the underlying metabolic flux distribution.

### 3.4 Carbon flux distribution in DHB producer strains

**Fig. 5:**
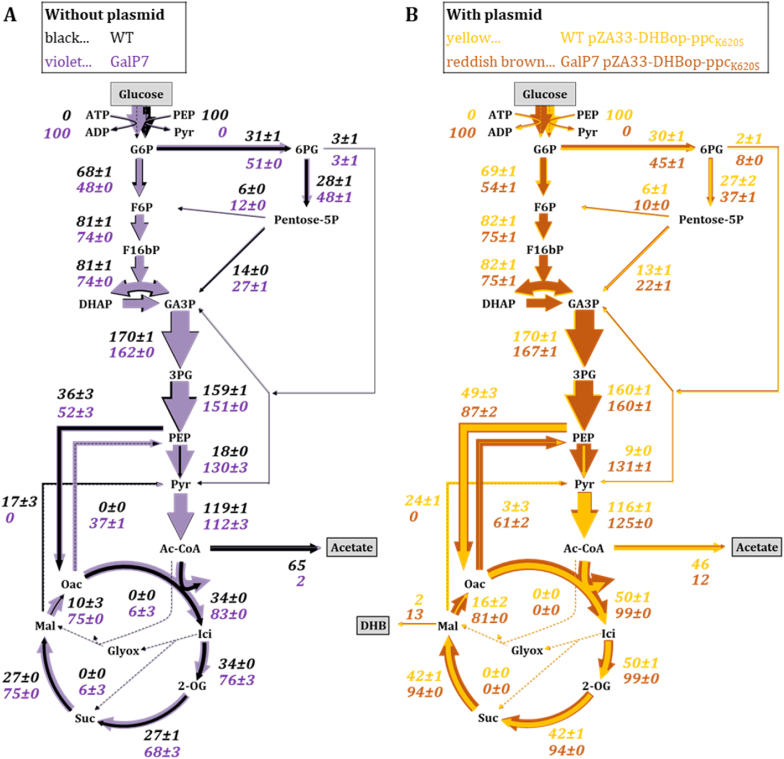
Carbon flux distribution of the wild type (black) and GalP7 (*ΔptsI* ΔP_galP_::P_proD_*ΔmaeA ΔmaeB ΔfrdBC ΔfumB ΔfumC*, violet) strains (A) and the strains harboring the pZA33-DHBop-ppc_K620S_ plasmid in the wild type (yellow) and GalP7 (reddish brown) backgrounds (B). Cultivations were carried out in 10 mL M9 supplemented with 2.5 g L^−1^ (1:4 of U-^13^C to 1-^13^C) glucose and 1 mM IPTG. Fluxes are indicated as mol percent per consumed glucose. Flux values represent the mean of two biological replicates, and the indicated variation reflects the standard deviation between replicates. Thickness of the arrows is proportional to carbon flux values. DHB and acetate were calculated from measured concentrations in the supernatant. Abbreviations: AcCoA – Acetyl-CoA; *α*-KG – *α*-Ketoglutarate; F6P – fructose-6-phosphate, F16bP – fructose-1,6-bis-phosphate; Fum – Fumarate; G6P – glucose-6-phosphate; GA3P – glyceraldehyde-3-phosphate; Ici – Isocitrate; Mal – Malate; OAA – Oxaloacetate; PEP – Phosphoenolpyruvate; 3 PG – 3-phosphoglycerate; PYR – Pyruvate; Suc – Succinate.

To uncover remaining metabolic limitations for efficient DHB production, we performed ^13^C-MFA. The analysis included the GalP7 and WT strains, each evaluated with (w.P) and without (w/o.P) the DHB production plasmid pZA33-DHBop-ppc_K620S_ (Fig. 5). This setup allows us to distinguish between metabolic changes caused by host strain engineering, such as PTS deletion and malate/fumarate pathway inactivation, and effects resulting from the expression of the DHB pathway including the malate-insensitive Ppc_K620S_ enzyme. The strains were cultivated in M9 medium supplemented with 2.5 g L^−1^ (1:4 of U-^13^C to 1-^13^C) glucose. Flux distributions were estimated from labeling patterns of proteinogenic amino acids (Supplementary File 2) and extracellular yields (Table 2). The goodness-of-fit based on the χ^2^ values indicated good agreement between measured and simulated labeling patterns (Supplementary File 1 Table S4).

**Table 2:** Yields (Y) of DHB, Acetate (Ac) and biomass (BM) calculated from the consumed glucose (Glc_up_) and product concentrations. The values were used for the determination of the carbon flux distributions with and without DHB production plasmid expressed from the pZA33 vector.

Strain	Glc_up_ [g L^−1^]	DHB [g L^−1^]	Ac [g L^−1^]	Y_DHB_ [mol mol^−1^]	Y_Ac_ [mol mol^−1^]	Y_BM_ [g mmol^−1^]
WT	1.19 ± 0.08	-	0.26 ± 0.01	-	0.65 ± 0.02	0.07 ± 0.00
GalP7	0.99 ± 0.01	-	0.01 ± 0.00	-	0.02 ± 0.00	0.07 ± 0.00
WT pZA33-DHBop-ppc_K620S_	1.19 ± 0.04	0.02 ± 0.00	0.18 ± 0.00	0.02 ± 0.00	0.46 ± 0.02	0.07 ± 0.00
GalP7 pZA33-DHBop-ppc_K620S_	1.77 ± 0.10	0.15 ± 0.01	0.07 ± 0.02	0.13 ± 0.00	0.12 ± 0.03	0.05 ± 0.00

For the WT w/o.P (Fig. 5A, black colored arrows), fluxes were largely consistent with previous studies (Sauer et al., 2004), except for elevated malic enzyme flux and higher acetate secretion (0.65 mol mol^−1^, Table 2; Supplementary File 1 Table S5). On the other hand, the acetate formation in the GalP7 w/o.P was almost abolished with a yield of 0.02 mol mol^−1^ (Table 2). For calculation of the carbon distribution in the GalP7 strains, the metabolic network was further constrained by excluding PTS-mediated glucose uptake and malic enzyme activities. Compared to the WT w/o.P, the flux distributions in GalP7 w/o.P showed higher activity in the pentose phosphate pathway, the TCA cycle, and the Ppc reaction (Fig. 5A, violet colored arrows). The forward flux from PEP to oxaloacetate increased from 36 to 52 mol-%, but the reverse flux via Pck reduced the net contribution to 17 mol-%. Due to the reversible nature of the conversion between PEP/Pck node, individual forward and backward fluxes should be interpreted with caution, as these reactions are tightly coupled, whereas the resulting net flux between PEP and oxaloacetate is more robustly determined. Despite this limitation, the observed increase in reverse flux is consistent with earlier studies reporting elevated Pck activity in PTS-negative strains overexpressing *galP* (Flores et al., 2002; Meza et al., 2012).

Expression of the DHB pathway in the WT background (WT w.P) resulted in a DHB yield of 0.02 mol mol^−1^ and acetate production of 0.46 mol mol^−1^ (Table 2). This coincided with an increased TCA cycle flux to 50 mol-% (Fig. 5A, black colored arrows) compared to 37 mol-% in the WT w/o.P (Fig. 5B, yellow colored arrows). The expression of the mutant *ppc*_*K620S*_ further increased overall Ppc flux to 49 mol-% at the expense of the Pyk reaction (9 mol-%) (Fig. 5B, yellow colored arrows). The increase in TCA cycle and Ppc forward flux was also observed when comparing the GalP7 w/o.P and GalP7 w.P strains (Fig. 5B, reddish-brown colored arrows). GalP7 w.P displayed the highest TCA cycle flux in this study (99 mol-%), even though acetate production increased from 0.02 to 0.12 mol mol^−1^ (Table 2). Overexpression of *ppc*_*K620S*_ further enhances the forward reaction from PEP to oxaloacetate from initially 52 to 87 mol-% (w/o.P vs w.P). But this shift is partly compensated by an increased backward flux (61 mol-%), resulting in a net flux of 26 mol-% in GalP7 w.P.

Overall, the GalP7 strain displayed increased TCA cycle activity, an enhanced forward anaplerotic flux from PEP via Ppc, and reduced acetate overflow. The increased flux through the pentose phosphate pathway suggests improved NADPH availability, which could be favorable for the NADPH-dependent reactions of the DHB pathway (Fig. 1). We did not observe a split of the TCA cycle with net-carbon flux from OAA to DHB via the reverse malate dehydrogenase reaction. Instead, all OAA was condensed with acetyl-CoA to yield citrate and channeled into the TCA cycle.

### 3.5 Further metabolic requirements for optimized DHB production in PTS-deficient strains

**Fig. 6:**
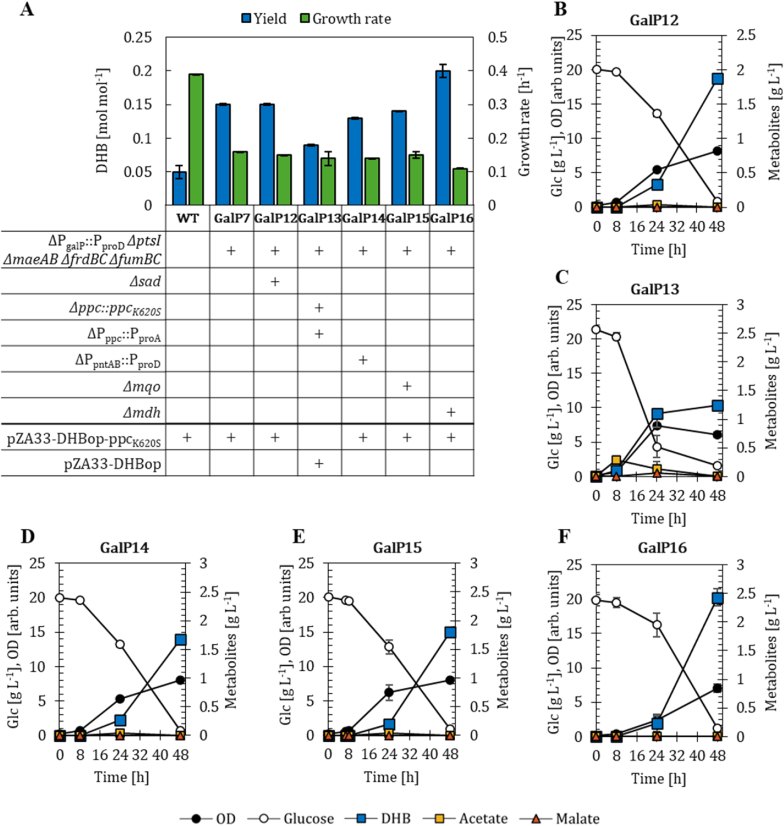
DHB yields after 48 h of cultivation and growth rates of GalP7 (ΔP_galP_::P_proD_*ΔptsI ΔmaeA ΔmaeB ΔfrdBC ΔfumB ΔfumC*)-derived host strains (A). Strains are harbouring the DHB production plasmid pZA33-DHBop-ppc_K620S_. Corresponding fermentation profiles in M9 minimal medium supplemented with 20 g L^−1^ of glucose are shown for: GalP7 *Δsad* (GalP12, B), GalP7 *Δppc::ppc*_*K620S*_ ΔP_ppc_::P_proA_ (GalP13, C), GalP7 ΔP_pntAB_::P_proD_ (GalP14, D), GalP7 *Δmqo* (GalP15, E) and GalP7 *Δmdh* (GalP16, F). In (A) deletion (Δ) of target genes is marked with a (+) sign; in (B-E) mean values are shown as colored symbols. All results correspond to the mean of at least two biological replicates with error bars representing the standard deviation of the mean (see Supplementary File 1 Table S3).

We further investigated other metabolic requirements for improved DHB production using the PTS-deficient host strain GalP7 as the chassis. In our previous study, we discovered that the inactivation of succinic semialdehyde dehydrogenase Sad enhanced DHB production by three-fold to 0.15 mol mol^−1^ (Nguyen et al., 2025). We proposed that Sad was responsible for futile conversion of the intermediate malate semialdehyde back to malate. On this basis, we introduced the *sad* deletion into the GalP7 strain. In contrast to our expectations, the resulting host strain GalP12 did not produce DHB at higher yields compared to GalP7. Both strains exhibited a DHB yield of 0.15 mol mol^−1^ when expressing the plasmid pZA33-DHBop-ppc_K620S_ (Fig. 6A; Supplementary File 1 Table S3). This suggests that the inactivation of Sad is not a metabolic requirement for optimized DHB production in PTS-deficient strains. Acetate or other byproducts could not be detected in the culture supernatant (Fig. 6B).

The modulation of the malate-insensitive Ppc_K620S_ enzyme was previously identified as important as well. Therefore, we replaced the chromosomally expressed *ppc* gene and its respective canonical promoter with the *ppc*_*K620S*_ mutant preceded by the weak, constitutive proA promoter (Davis et al., 2011) in the GalP7 host (GalP13), to fine-tune anaplerotic flux and avoid potential metabolic imbalances caused by excessive Ppc activity. This configuration has previously enabled improved DHB titers in both the WT and the sad mutant using the smaller pZA33-DHBop plasmid, which omits the *ppc*_*K620S*_ gene and therefore exhibits higher plasmid stability (Nguyen et al., 2025). When expressing this plasmid in the GalP13 host, a DHB yield of 0.09 mol mol^−1^ was observed, and acetate accumulated within the first 24 h of cultivation (Fig. 6C). Compared to the GalP7 strain (expressing pZA33-DHBop-ppc_K620S_) we observed a 40 % reduction in DHB yield, which suggests that altering the promoter strength of the *ppc* gene can negatively impact carbon flux towards DHB.

The previous ^13^C-MFA of GalP7 (Fig. 5A and B) revealed an increased flux through the pentose phosphate pathway, suggesting elevated NADPH availability compared to the WT (Yuan et al., 2022). Because the malate semialdehyde dehydrogenase and malate semialdehyde reductase of the DHB pathway (Fig. 1) are NADPH-dependent, we tested whether additional NADPH supply could further enhance DHB production. To this end, the proton-translocating transhydrogenase encoding genes *pntAB*, to catalyze the reversible transfer of hydride ions from NADH to NADP^+^ to form NADPH and NAD^+^, were overexpressed under the strong, constitutive proD promoter, generating strain GalP14 (Fig. 6D). However, in the GalP7 background, *pntAB* overexpression resulted in a DHB yield of 0.13 mol mol^−1^, indicating that under our experimental conditions additional NADPH supply did not significantly enhance DHB production, and therefore NADPH availability is unlikely to be strongly limiting. Based on the flux distribution obtained from ^13^C-MFA, a simplified stoichiometric NADPH balance considering pentose phosphate pathway derived NADPH formation and DHB synthesis as the NADPH sink also suggests that NADPH supply is sufficient to support DHB production.

The ^13^C-MFA also revealed an elevated flux from malate to oxaloacetate, in the GalP7 strains which had the malic enzymes deleted (Fig. 5). We therefore aimed to reduce malate dehydrogenase activity to increase the malate pool and promote DHB formation. To test this, we investigated the deletion of either the membrane-bound malate dehydrogenase gene *mqo* (GalP15) or the cytosolic malate dehydrogenase gene *mdh* (GalP16). Deletion of *mqo* had a negligible effect on DHB yield (0.14 mol mol^−1^ compared to 0.15 mol mol^−1^ in the reference strain GalP7), whereas deletion of *mdh* increased it to 0.20 mol mol^−1^ (Fig. 6E and F). This indicates that in the GalP7 background cytosolic malate oxidation via Mdh plays a dominant role in directing malate toward oxaloacetate, whereas Mqo contributes only marginally under these conditions. Deletion of *mdh* reduces the conversion of oxidation of malate to oxaloacetate and increases the intracellular availability of malate for DHB formation. The increase in DHB yield upon *mdh* deletion further suggests that Mqo cannot fully compensate for the loss of Mdh activity.

Overall, the results demonstrate that limiting Mdh-dependent malate oxidation is critical for optimal DHB production in PTS-deficient strains, while an additional NADPH supply or chromosomal expression of the *ppc*_*K620S*_ gene encoding for the malate-insensitive Ppc mutant provides no further benefit in the GalP7 background.

## 4 Discussion and conclusion

In this study, we engineered *E. coli* strains for efficient DHB production by implementing the alternative GalP/Glk system as the primary route for glucose uptake. Replacement of the PTS system with the GalP transporter, in combination with the sequential inactivation of reactions, which consume malate or which are implicated in the TCA cycle (*ΔmaeA ΔmaeB ΔfrdBC ΔfumB ΔfumC*), yielded a strain capable of producing DHB at a yield of 0.15 mol mol^−1^ without detectable byproduct formation. This demonstrates that redirecting carbon flux from catabolic overflow and TCA-associated side branches can effectively enhance flux toward DHB formation.

Compared with PTS-active strains, which secreted and subsequently reassimilated acetate, the PTS-deficient background maintained a balanced flux between glycolysis and the TCA cycle, thereby avoiding acetate overflow (Veit et al., 2007; De Anda et al., 2006). Such behavior is typical for PTS-deficient *E. coli* strains and is generally associated with reduced carbon loss and improved redox homeostasis. The decoupling of growth and product formation in these strains suggests that PEP conservation and increased forward anaplerotic input through Ppc contributes to the observed redirection of carbon toward DHB.

Among the targeted reactions, deletion of fumarate reductase Frd resulted in the largest increase in DHB yield. Although Frd is typically inactive under aerobic conditions (Jones and Gunsalus, 1987), our results indicate that its activity can be functionally relevant when the *galP* gene is overexpressed. The deletion of *frdBC* may have prevented futile cycling between fumarate and succinate, thereby increasing the net flux through the oxidative TCA branch. Further transcriptional or enzymatic analyses would be required to clarify this mechanism.

Contrary to our expectations, inactivation of the malic enzymes MaeA and MaeB only increased DHB yield in the PTS-deficient background. Under PTS-active conditions, deletion of *maeA* and *maeB* reduced DHB production and led to the accumulation of malate when Frd was additionally inactivated. This indicates that, under PTS-active conditions, increased malate availability does not translate into higher DHB production. Instead, it might have revealed a limitation in the downstream conversion of malate to DHB. In contrast, in the PTS-deficient background, malate availability appears to be more effectively coupled to DHB synthesis. Together, these observations indicate that, under PTS-active conditions, increasing malate availability alone does not appear to be sufficient to enhance DHB production. Rather, efficient partitioning of flux at the malate node and sufficient downstream pathway capacity are required to direct carbon toward DHB. This context-dependent behavior emphasizes the importance of balancing precursor generation with pathway demand when optimizing DHB-producing strains.

Interestingly, the beneficial effect of *sad* deletion, which previously improved DHB yields threefold in PTS-positive backgrounds (Nguyen et al., 2025), was absent in the PTS-deficient strain. ^13^C-MFA data indicated an enhanced flux through the pentose phosphate (PP) pathway in the GalP7 strain, suggesting an increased NADPH supply (Yuan et al., 2022; Poulsen et al., 2005). As the conversion of malate semialdehyde to DHB is NADPH-dependent, higher NADPH availability may have prevented accumulation of malate semialdehyde, rendering Sad inactivation unnecessary.

Flux analysis showed a strong forward flux from PEP to oxaloacetate via Ppc, even though the increased backward reaction, presumably catalyzed by Pck, decreased the overall anaplerotic net flux. However, chromosomal expression of the malate-insensitive *ppc*_*K620S*_ variant from a moderate promoter reduced DHB yield, indicating that the expression level of *ppc* was not well aligned with the metabolic requirements of the PTS-negative GalP7 background (Shi et al., 2017; De Maeseneire et al., 2006; Peng et al., 2004). In addition, deletion of *mdh* further improved DHB yield to 0.20 mol mol^−1^, confirming that excessive oxidation of malate to oxaloacetate competes with DHB formation. Malate is generated via the oxidative TCA cycle and subsequently oxidized to oxaloacetate by Mdh under aerobic conditions (Unden and Kleefeld, 2004). Therefore, deletion of *mdh* increases the availability of malate for DHB formation, while Mqo appears unable to compensate for the loss of Mdh activity. Deletion of *mqo* did not increase DHB yield, suggesting that Mqo contributes less to malate oxidation under the tested conditions, possibly due to a lower flux through this reaction. This difference may be related to the distinct cofactor usage of the two enzymes, as Mdh is NAD^+^-dependent whereas Mqo is coupled to the quinone pool. Together, these results highlight the importance of maintaining a sufficient malate pool to sustain efficient DHB production.

Future improvements may target the fine-tuning of flux distribution between anaplerotic and oxidative TCA reactions—for instance, by limiting the reverse flux via Pck or activating the glyoxylate shunt through *iclR* deletion or *aceAK* overexpression (Deng et al., 2018; Kornberg, 1966). Furthermore, optimization of the DHB pathway enzymes themselves could enhance conversion efficiency under high TCA-flux conditions.

**Fig. 7:**
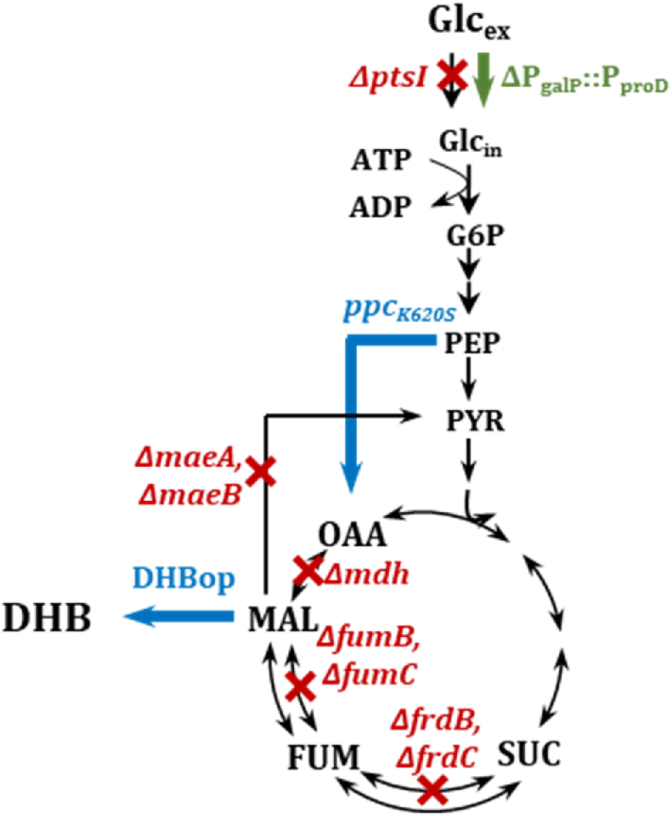
Schematic representation of the optimized *E. coli* MG1655 strain GalP16 for efficient DHB production. Red crosses indicate targeted gene deletions (*ΔptsI, ΔmaeA, ΔmaeB, ΔfrdB, ΔfrdC, ΔfumB, ΔfumC, Δmdh*). Green arrows represent overexpression via chromosomal promoter exchange (ΔP_galP_::P_proD_). Blue arrows indicate overexpression of the DHB pathway (DHBop) and the *ppc*_*K620S*_ gene, encoding a malate-insensitive variant, from the DHB production plasmid pZA33-DHBop-ppc_K620S_. Abbreviations: FUM – Fumarate; DHB – 2,4-Dihydroxybutyric acid; Glc_ex_ – Extracellular glucose; Glc_in_ – Intracellular glucose; G6P – Glucose-6-phosphate; MAL – Malate; OAA – Oxaloacetate; PEP – Phosphoenolpyruvate; PYR – Pyruvate.

In this context, the achieved DHB yield of 0.20 mol mol^−1^ (GalP16; Fig. 7) corresponds to approximately 15% of the theoretical maximum yield of 1.33 mol mol^−1^ under aerobic conditions (Wagner et al., 2023b), indicating that substantial potential for further metabolic and process optimization remains. Nevertheless, the GalP-based PTS-deficient background provides a robust metabolic chassis for DHB biosynthesis. The reduced acetate overflow, enhanced TCA and PP pathway activities, and improved redox balance together are favorable for NADPH-dependent DHB formation from the TCA-intermediate malate. These features make the GalP7-derived strains promising candidates for further metabolic optimization and scale-up to controlled bioreactor processes.

## CRediT authorship contribution statement

**T.A. Stefanie Nguyen:** Writing – review & editing, Writing – original draft, Visualization, Methodology, Investigation, Formal analysis, Data curation. **Ceren Alkim:** Investigation. **Nadine Ihle:** Investigation. **Thomas Walther:** Writing – review & editing, Supervision, Project administration, Funding acquisition, Conceptualization. **Cláudio J.R. Frazão:** Writing – review & editing, Supervision, Methodology, Investigation, Conceptualization.

## Declaration of competing interest

The authors declare that they have no known competing financial interests or personal relationships that could have appeared to influence the work reported in this paper.

## Data Availability

Data will be made available on request.

## References

[bib1] Baba T., Ara T., Hasegawa M., Takai Y., Okumura Y., Baba M., Datsenko K.A., Tomita M., Wanner B.L., Mori H. (2006). Construction of *Escherichia coli* K‐12 in‐frame, single‐gene knockout mutants: the Keio collection. Mol. Syst. Biol..

[bib2] Chauvin F., Brand L., Roseman S. (1996). Enzyme I: the first protein and potential regulator of the bacterial phosphoenolpyruvate: glycose phosphotransferase system. Res. Microbiol..

[bib3] Cherepanov P.P., Wackernagel W. (1995). Gene disruption in *Escherichia coli*: TcR and KmR cassettes with the option of Flp-catalyzed excision of the antibiotic-resistance determinant. Gene.

[bib4] Chubukov V., Gerosa L., Kochanowski K., Sauer U. (2014). Coordination of microbial metabolism. Nat. Rev. Microbiol..

[bib5] Datsenko K.A., Wanner B.L. (2000). One-step inactivation of chromosomal genes in *Escherichia coli* K-12 using PCR products. Proc. Natl. Acad. Sci. USA.

[bib6] Davis J.H., Rubin A.J., Sauer R.T. (2011). Design, construction and characterization of a set of insulated bacterial promoters. Nucleic Acids Res..

[bib7] De Anda R., Lara A.R., Hernández V., Hernández-Montalvo V., Gosset G., Bolívar F., Ramírez O.T. (2006). Replacement of the glucose phosphotransferase transport system by galactose permease reduces acetate accumulation and improves process performance of *Escherichia coli* for recombinant protein production without impairment of growth rate. Metab. Eng..

[bib8] De Maeseneire S.L., De Mey M., Vandedrinck S., Vandamme E.J. (2006). Metabolic characterisation of E. coli citrate synthase and phosphoenolpyruvate carboxylase mutants in aerobic cultures. Biotechnol. Lett..

[bib9] Deng Y., Ma N., Zhu K., Mao Y., Wei X., Zhao Y. (2018). Balancing the carbon flux distributions between the TCA cycle and glyoxylate shunt to produce glycolate at high yield and titer in *Escherichia coli*. Metab. Eng..

[bib10] Flores S., Gosset G., Flores N., DeGraaf A.A., Bolivar F. (2002). Analysis of carbon metabolism in *Escherichia coli* strains with an inactive phosphotransferase system by 13C labeling and NMR spectroscopy. Metab. Eng..

[bib11] Fraenkel D.G., Falcoz-Kelly F., Horecker B.L. (1964). The utilization of glucose 6-phosphate by glucokinaseless and wild-type strains of *Escherichia coli*. Proc. Natl. Acad. Sci..

[bib12] François J.M. (2023). Progress advances in the production of bio-sourced methionine and its hydroxyl analogues. Biotechnol. Adv..

[bib13] Frazão C.J.R., Topham C.M., Malbert Y., François J.M., Walther T. (2018). Rational engineering of a malate dehydrogenase for microbial production of 2,4-dihydroxybutyric acid via homoserine pathway. Biochem. J..

[bib14] Frazão C.J.R., Trichez D., Serrano-Bataille H., Dagkesamanskaia A., Topham C.M., Walther T., François J.M. (2019). Construction of a synthetic pathway for the production of 1,3-propanediol from glucose. Sci. Rep..

[bib15] Frazão C.J.R., Wagner N., Rabe K., Walther T. (2023). Construction of a synthetic metabolic pathway for biosynthesis of 2,4-dihydroxybutyric acid from ethylene glycol. Nat. Commun..

[bib16] Fuentes L.G., Lara A.R., Martínez L.M., Ramírez O.T., Martínez A., Bolívar F., Gosset G. (2013). Modification of glucose import capacity in *Escherichia coli*: physiologic consequences and utility for improving DNA vaccine production. Microb. Cell Fact..

[bib17] Green M.R., Sambrook J. (2012).

[bib18] Henderson P.J.F., Giddens R.A., Jones-Mortimer M.C. (1977). Transport of galactose, glucose and their molecular analogues by *Escherichia coli* K12. Biochem. J..

[bib19] Ihle N., Grüßner L., Alkim C., Nguyen T.A.S., Walther T., Frazão C.J.R. (2025). Cofactor engineering for improved production of 2,4-dihydroxybutyric acid via the synthetic homoserine pathway. Front. Bioeng. Biotechnol..

[bib20] Jones H.M., Gunsalus R.P. (1987). Regulation of *Escherichia coli* fumarate reductase (frdABCD) operon expression by respiratory electron acceptors and the fnr gene product. J. Bacteriol..

[bib21] Kornberg H.L. (1966). The role and control of the glyoxylate cycle in *Escherichia coli*. Biochem. J..

[bib22] Lennox E.S. (1955). Transduction of linked genetic characters of the host by bacteriophage P1. Virology.

[bib23] Li X., Cai Z., Li Y., Zhang Y. (2014). Design and construction of a non-natural malate to 1,2,4-Butanetriol pathway creates possibility to produce 1,2,4-Butanetriol from glucose. Sci. Rep..

[bib24] Liu Y., Zhang J., Li R., Yu B. (2022). Efficient production of 2,4-Dihydroxybutyrate from l -Homoserine by the designed cofactor self-sufficient route. ACS Sustainable Chem. Eng..

[bib25] Meza E., Becker J., Bolivar F., Gosset G., Wittmann C. (2012). Consequences of phosphoenolpyruvate:sugar phosphotranferase system and pyruvate kinase isozymes inactivation in central carbon metabolism flux distribution in *Escherichia coli*. Microb. Cell Fact..

[bib26] Millard P., Enjalbert B., Uttenweiler-Joseph S., Portais J.-C., Létisse F. (2021). Control and regulation of acetate overflow in *Escherichia coli*. eLife.

[bib27] Millard P., Letisse F., Sokol S., Portais J.-C. (2012). IsoCor: correcting MS data in isotope labeling experiments. Bioinformatics.

[bib28] Nguyen T.A.S., Alkim C., Ihle N., Walther T., Frazão C.J.R. (2025). Deletion of succinic semialdehyde dehydrogenase sad and chromosomal expression of phosphoenolpyruvate carboxylase as metabolic requirements for improved production of 2,4-dihydroxybutyric acid via malyl-P pathway using E. coli. Front. Bioeng. Biotechnol..

[bib29] Peng L., Arauzo-Bravo M.J., Shimizu K. (2004). Metabolic flux analysis for a ppc mutant *Escherichia coli* based on 13C-labelling experiments together with enzyme activity assays and intracellular metabolite measurements. FEMS Microbiol. Lett..

[bib30] Poulsen B.R., Nøhr J., Douthwaite S., Hansen L.V., Iversen J.J.L., Visser J., Ruijter G.J.G. (2005). Increased NADPH concentration obtained by metabolic engineering of the pentose phosphate pathway in *Aspergillus niger*. FEBS J..

[bib47] Sauer U., Canonaco F., Heri S., Perrenoud A., Fischer E. (2004). The Soluble and Membrane-bound Transhydrogenases UdhA and PntAB Have Divergent Functions in NADPH Metabolism of Escherichia coli. J. Biol. Chem..

[bib31] Shi F., Zhang M., Li Y. (2017). Overexpression of ppc or deletion of mdh for improving production of γ-aminobutyric acid in recombinant Corynebacterium glutamicum. World J. Microbiol. Biotechnol..

[bib32] Sokol S., Millard P., Portais J.-C. (2012). Influx_s: increasing numerical stability and precision for metabolic flux analysis in isotope labelling experiments. Bioinformatics.

[bib33] Steinsiek S., Bettenbrock K. (2012). Glucose transport in *Escherichia col*i mutant strains with defects in sugar transport systems. J. Bacteriol..

[bib34] Trichez D., Auriol C., Baylac A., Irague R., Dressaire C., Carnicer-Heras M., Heux S., François J.M., Walther T. (2018). Engineering of *Escherichia coli* for Krebs cycle-dependent production of malic acid. Microb. Cell Fact..

[bib35] Tseng C.-P., Yu C.-C., Lin H.-H., Chang C.-Y., Kuo J.-T. (2001). Oxygen- and growth rate-dependent regulation of *Escherichia coli* fumarase (FumA, FumB, and FumC) activity. J. Bacteriol..

[bib36] Unden G., Kleefeld A. (2004). C_4_ -Dicarboxylate degradation in aerobic and anaerobic growth. EcoSal Plus.

[bib37] Veit A., Polen T., Wendisch V.F. (2007). Global gene expression analysis of glucose overflow metabolism in *Escherichia coli* and reduction of aerobic acetate formation. Appl. Microbiol. Biotechnol..

[bib38] Vemuri G.N., Altman E., Sangurdekar D.P., Khodursky A.B., Eiteman M.A. (2006). Overflow metabolism in *Escherichia coli* during steady-state growth: transcriptional regulation and effect of the redox ratio. Appl. Environ. Microbiol..

[bib39] Wagner N., Bade F., Straube E., Rabe K., Frazão C.J.R., Walther T. (2023). In vivo implementation of a synthetic metabolic pathway for the carbon-conserving conversion of glycolaldehyde to acetyl-CoA. Front. Bioeng. Biotechnol..

[bib40] Wagner N., Wen L., Frazão C.J.R., Walther T. (2023). Next-generation feedstocks methanol and ethylene glycol and their potential in industrial biotechnology. Biotechnol. Adv..

[bib41] Walther T., Calvayrac F., Malbert Y., Alkim C., Dressaire C., Cordier H., François J.M. (2018). Construction of a synthetic metabolic pathway for the production of 2,4-dihydroxybutyric acid from homoserine. Metab. Eng..

[bib42] Walther T., Topham C.M., Irague R., Auriol C., Baylac A., Cordier H., Dressaire C., Lozano-Huguet L., Tarrat N., Martineau N., Stodel M., Malbert Y., Maestracci M., Huet R., André I., Remaud-Siméon M., François J.M. (2017). Construction of a synthetic metabolic pathway for biosynthesis of the non-natural methionine precursor 2,4-dihydroxybutyric acid. Nat. Commun..

[bib43] Yuan L., Qin Y.-L., Zou Z.-C., Appiah B., Huang H., Yang Z.-H., Qun C. (2022). Enhancing intracellular NADPH bioavailability through improving pentose phosphate pathway flux and its application in biocatalysis asymmetric reduction reaction. J. Biosci. Bioeng..

[bib46] Zhang X., Wang X., Shanmugam K.T., Ingram L.O. (2011). L-malate production by metabolically engineered *Escherichia**coli*. Appl. Environ. Microbiol..

[bib44] Zhang J., Wang Y., Wang D., Yu B. (2025). Fermentative production of 2,4-Dihydroxybutyrate from glucose via a Redox balanced route. ACS Sustainable Chem. Eng..

[bib45] Zhang X., Jantama K., Moore J.C., Jarboe L.R., Shanmugam K.T., Ingram L.O. (2009). Metabolic evolution of energy-conserving pathways for succinate production in *Escherichia coli*. Proc. Natl. Acad. Sci. USA.

