# Supplementary material for: Engineering of the glucose uptake system to increase 2,4-Dihydroxybutyric acid production in Escherichia coli
Source: Metab Eng Commun. 2026 Apr 15;22:e00276. doi: 10.1016/j.mec.2026.e00276 (PMC13101720; doi:10.1016/j.mec.2026.e00276)
Supplement: Multimedia component 1 [file mmc1.docx]

Engineering of the glucose uptake system to increase 2,4-Dihydroxybutyric acid production in *Escherichia coli*

*Supplementary Material*

**T.A. Stefanie Nguyen^1^, Ceren Alkim^2^, Nadine Ihle^1^, Thomas Walther^1*^, Cláudio J.R. Frazão^1^**

^1^Chair of Bioprocess Engineering, Institute of Natural Materials Technology, TU Dresden, Bergstraße 120, 01062 Dresden, Germany

^2^Toulouse Biotechnology Institute, UMR INSA-CNRS5504 and UMR INSA-INRAE 792; 135 avenue de Rangueil, 31077 Toulouse, France

***Correspondence:**Prof. Thomas Walther
Email: [thomas_walther@tu-dresden.de](mailto:thomas_walther@tu-dresden.de)

Table S1 Fermentation products of DHB, acetate (Ac), malate (Mal), corresponding yields (Y) and growth rates (µ) of **PTS-inactive and *galP* overexpressing** host srains harbouring the DHB production plasmid pZA33-DHBop-ppc_K620S_. Results after 48 hours of cultivation in M9 mineral medium with 20 g L^-1^ glucose are shown.

| **Host**  **strain** | **Characteristics** | **DHB**  **[g L^-1^]** | **Ac**  **[g L^-1^]** | **Mal**  **[g L^-1^]** | **Y_DHB**  **[mol mol^-1^]** | **Y_Ac**  **[mol mol^-1^]** | **Y_Mal**  **[mol mol^-1^]** | **µ**  **[h^-1^]** |
| --- | --- | --- | --- | --- | --- | --- | --- | --- |
| WT | *E. coli* K-12 MG1655 | 0.60±0.06 | - | - | 0.05±0.01 | - | - | 0.39±0.03 |
| GalP0 | *∆ptsI* | - | - | - | - | - | - | 0.03±0.01 |
| GalP1 | GalP1 *∆ptsI* | 0.58±0.06 | 0.13±0.13 | - | 0.04±0.00 | 0.02±0.02 | - | 0.09±0.01 |
| GalP2 | ∆P_galP_::P_proD_ | 1.19±0.01 | - | - | 0.09±0.00 | - | - | 0.34±0.00 |
| GalP3 | GalP2 *∆maeA* | 0.50±0.12 | - | - | 0.04±0.01 | - | - | 0.10±0.00 |
| GalP4 | GalP3 *∆maeB* | 1.01±0.07 | - | - | 0.08±0.01 | - | - | 0.14±0.01 |
| GalP5 | GalP4 *∆frdBC* | 1.76±0.10 | - | - | 0.13±0.01 | - | - | 0.13±0.01 |
| GalP6 | GalP5 *∆fumB* | 1.86±0.12 | 0.07±0.07 | - | 0.14±0.00 | 0.01±0.01 | - | 0.16±0.01 |
| GalP7 | GalP6 *∆fumC* | 1.94±0.02 | - | - | 0.15±0.00 | - | - | 0.16±0.00 |

Table S2 Fermentation products of DHB, acetate (Ac), malate (Mal), corresponding yields (Y) and growth rates (µ) **of PTS-active and *galP* overexpressing** srains harbouring the DHB production plasmid pZA33-DHBop-ppc_K620S_. Results after 24 hours of cultivation in M9 mineral medium with 20 g L^-1^ glucose are shown.

| **Host**  **strain** | **Characteristics** | **DHB**  **[g L^-1^]** | **Ac**  **[g L^-1^]** | **Mal**  **[g L^-1^]** | **Y_DHB**  **[mol mol^-1^]** | **Y_Ac**  **[mol mol^-1^]** | **Y_Mal**  **[mol mol^-1^]** | **µ**  **[h^-1^]** |
| --- | --- | --- | --- | --- | --- | --- | --- | --- |
| GalP1 | ∆P_galP_::P_proD_ | 1.19±0.01 | - | - | 0.09±0.00 | - | - | 0.34±0.00 |
| Frd1 | *∆frdBC* | 0.56±0.06 | - | - | 0.04±0.01 | - | - | 0.39±0.00 |
| GalP8 | GalP1 *∆frdBC* | 1.46±0.06 | 0.04±0.04 | - | 0.12±0.00 | 0.01±0.01 | - | 0.33±0.01 |
| GalP9 | GalP8 *∆maeA ∆maeB* | 0.84±0.00 | 0.05±0.00 | 1.61±0.34 | 0.06±0.00 | 0.01±0.00 | 0.11±0.02 | 0.31±0.01 |
| GalP10 | GalP9 *∆fumB* | 0.92±0.02 | 0.56±0.21 | 0.12±0.01 | 0.07±0.00 | 0.09±0.03 | 0.01±0.00 | 0.34±0.00 |
| GalP11 | GalP10 *∆fumC* | 0.85±0.06 | 0.81±0.14 | 0.12±0.03 | 0.06±0.00 | 0.12±0.02 | 0.01±0.00 | 0.35±0.01 |

Table S3 Fermentation products of DHB, acetate (Ac), malate (Mal), corresponding yields (Y) and growth rates (µ) of **GalP7-derived host strains** harbouring the DHB production plasmid pZA33-DHBop-ppc_K620S_. Results after 48 hours of cultivation in M9 mineral medium with 20 g L^-1^ glucose are shown. The GalP7 host contains the modifications ∆P_galP_::P_proD_ *∆ptsI* *∆maeA ∆maeB ∆frdBC ∆fumB ∆fumC*.

| **Background** | | **Characteristics** | | **DHB**  **[g L^-1^]** | | **Ac**  **[g L^-1^]** | | **Mal**  **[g L^-1^]** | | **Y_DHB**  **[mol mol^-1^]** | | **Y_Ac**  **[mol mol^-1^]** | | **Y_Mal**  **[mol mol^-1^]** | | **µ**  **[h^-1^]** | |
| --- | --- | --- | --- | --- | --- | --- | --- | --- | --- | --- | --- | --- | --- | --- | --- | --- | --- |
| GalP12 | | GalP7 *∆sad* | | 1.88±0.02 | | - | | - | | 0.15±0.00 | | - | | - | | 0.15±0.00 | |
| GalP13 | GalP7 ∆ppc::ppc_K620S_ ∆P_ppc_::P_proA_ | | 1.25±0.02 | | - | | - | | 0.09±0.00 | | - | | - | | 0.14±0.02 | |  |
| GalP14 | GalP7 ∆P_pntAB_::P_proD_ | | 1.68±0.05 | | - | | - | | 0.13±0.00 | | - | | - | | 0.14±0.00 | |  |
| GalP15 | GalP7 ∆P_galP_::P_proD_ ∆mqo | | 1.80±0.03 | | - | | - | | 0.14±0.00 | | - | | - | | 0.15±0.01 | |  |
| GalP16 | GalP7 ∆P_galP_::P_proD_ ∆mdh | | 2.43±0.13 | | - | | - | | 0.2±0.01 | | - | | - | | 0.11±0.00 | |  |

Table S4 Goodness-of-fit of ¹³C-metabolic flux analysis (MFA). χ² values were calculated by the *influx_s* software (version 5.3.0) based on the residuals between measured and simulated isotopologue distributions and were used to assess the agreement between experimental data and model predictions. Exp1 and Exp2 refer to independent biological replicates.

| **Strain** | **χ² Exp 1** | **χ² Exp 2** |
| --- | --- | --- |
| WT | 55.3 | 38.2 |
| GalP7 | 42.3 | 36.3 |
| WT pZA33-DHBop-ppc_K620S_ | 39.0 | 29.5 |
| GalP7 pZA33-DHBop-ppc_K620S_ | 67.1 | 50.0 |

Table S5 Comparison of growth rates and carbon fluxes for the most relevant pathways including Embden-Meyerhof-Parnas (EMP), Pentose phosphate (PP), Entner-Doudoroff (ED) and acetate production (Ac) pathway, as well as the TCA cycle and reactions catalysed by pyruvate knases (PYK), malate dehydrogenases (MDH), malic enzymes (MEZ), Ppc and pck enzymes. Concenstrained values are marked with an asterisk (*).

| **Strain** | **EMP** | **PP** | **PYK** | **TCA** | **MDH** | **Ppc** | **Pck** | **MEZ** | **Ac** | **µ [h^-1^]** |
| --- | --- | --- | --- | --- | --- | --- | --- | --- | --- | --- |
| MG1655 ^b)^ | 72 | 27 | 10 | 30 | 11 | 34 | 0 | 7 | 42 | 0.67 |
| JM101 ^c)^ | 77 | 22 | 41 | 44 | 38 | 25 | 12 | 0 | 55 | 0.71 |
| JM101 *∆ptsH ∆ptsI ∆crr* ^c)^ | 40 | 59 | 129 | 94 | 88 | 41 | 22 | 0 | 0 | 0.10 |
| W3110 ^d)^ | 83 | 16 | 64 | 21 | 18 | 23 | 8 | 0 | 117 | 0.69 |
| W3110 *∆ptsH ∆ptsI ∆crr* ∆P_galP_::P_Trc_ ^d)^ | 72 | 27 | 142 | 95 | 88 | 55 | 36 | 0 | 9 | 0.45 |
| MG1655 (WT) ^a)^ | 68 | 31 | 18 | 34 | 10 | 36 | 0 | 17 | 65* | 0.66 |
| GalP7 ^a)^ | 48 | 51 | 130 | 83 | 75 | 52 | 37 | 0* | 2* | 0.25 |
| WT pZA33-DHBop-ppc_K620S_ ^a)^ | 69 | 30 | 9 | 50 | 10 | 49 | 3 | 24 | 46* | 0.40 |
| GalP7 pZA33-DHBop-ppc_K620S_ ^a)^ | 54 | 45 | 131 | 99 | 75 | 87 | 61 | 0* | 12* | 0.12 |

^a)^ This study

^b)^ Sauer et al. (2004)

^c)^ Flores et al. (2002)

^d)^ Meza et al. (2012)

Table S6 Primers used in this study.

| **Primer** | **Sequence 5’ 🡪 3’** |
| --- | --- |
| *Knock-out* | |
| del_fumB-kanR_fw | GGCACGCCATTTTCGAATAACAAATACAGAGTTACAGGCTGGAAGCTATGGTGTAGGCTGGAGCTGCTTC |
| del_fumB-kanR_rev | GCATGCTGCCAGGCGCTGGGCCGAAGAGGTTACTTAGTGCAGTTCGCGCACATATGAATATCCTCCTTAG |
| *Verification* | |
| proD-galP_loc_fw | GCTGGCCTTTTTCTTTTGGATAG |
| proD-galP_loc_rv | ACCGATATCCAGGCCAAAGAG |
| ptsI_loc_fw | gtgacttccaacggcaaaag |
| ptsI_loc_rv | atagcaataccatcaccaacg |
| maeA(sfcA)_loc_fw | tagtaaataacccaaccggc |
| maeA(sfcA)_loc_rv | tacgtaacgtcaacatgatg |
| maeB_loc_fw | atggtattgctggattaagc |
| maeB_loc_rv | agatcactaagaatggagag |
| frdBC_loc_fw | gcgtctggacgaaggttgca |
| frdBC_loc_rv | caatgaagctctgcgcgaac |
| fumB_loc_fw | gattcagtttgaccgttccg |
| fumB_loc_rv | aaatgccggtataaatcagg |
| fumC_loc_fw | aggaaatgacttcttccag |
| fumC_loc_rv | tggtacaaaggagatcaaaa |
| sad_loc_fw | tagtgagattcgacggcacg |
| sad_loc_rv | cagatagcaatccccaatcg |
| proA-ppc_loc_rv | CTGGCAGCTTCGCCTTTC |
| proA-ppc_loc_fw | caccatttttgctggcattaa |
| ppc_loc1356_fw | GAAGTGCTCGATACCTGCCA |
| proD-pntAB_loc_rv | AAACGTGGCTGATTATTGC |
| proD-pntAB_loc_fw | CATCGAGCTTAGTGCGTCC |
| mqo_loc_rv | tatcagcatacgccacatccg |
| mqo_loc_fw | actgctgccgtcaggtcaat |
| mdh_loc_fw | GTTGGAATGTTGCGCTAATGC |
| mdh_loc_rv | GTATTCAGGTCAACGATCTG |
